# Towards evidence based strength training: a comparison of muscle forces during deadlifts, goodmornings and split squats

**DOI:** 10.1186/s13102-017-0077-x

**Published:** 2017-07-17

**Authors:** Florian Schellenberg, William R. Taylor, Silvio Lorenzetti

**Affiliations:** Institute for Biomechanics, HCP H 16.3, ETH Zurich, Leopold-Ruzicka-Weg 4, 8093 Zürich, Switzerland

**Keywords:** Musculoskeletal modelling, Muscle forces, Strength exercises, ACL

## Abstract

**Background:**

To ensure an efficient and targeted adaptation with low injury risk during strength exercises, knowledge of the participant specific internal loading conditions is essential. The goal of this study was to calculate the lower limb muscles forces during the strength exercises deadlifts, goodmornings and splits squats by means of musculoskeletal simulation.

**Methods:**

11 participants were assessed performing 10 different variations of split squats by varying the step length as well as the maximal frontal tibia angle, and 13 participants were measured performing deadlift and goodmorning exercises. Using individualised musculoskeletal models, forces of the Quadriceps *(*four parts), Hamstrings (four parts) and *m. gluteus maximus* (three parts) were computed.

**Results:**

Deadlifts resulted highest loading for the Quadriceps, especially for the vasti (18–34 N/kg), but not for the rectus femoris (8–10 N/kg), which exhibited its greatest loading during split squats (13–27 N/kg) in the rear limb. Hamstrings were loaded isometrically during goodmornings but dynamically during deadlifts. For the *m. gluteus maximus*, the highest loading was observed during split squats in the front limb (up to 25 N/kg), while deadlifts produced increasingly, large loading over large ranges of motion in hip and knee.

**Conclusions:**

Acting muscle forces vary between exercises, execution form and joint angle. For all examined muscles, deadlifts produced considerable loading over large ranges of motion, while split squats seem to be highly dependent upon exercise variation. This study provides key information to design strength-training programs with respect to loading conditions and ranges of motion of lower extremity muscles.

## Background

Resistance exercises are widely used in training programs and during rehabilitation to enhance performance, health and fitness through strengthening and adaptation of specific soft tissue and musculoskeletal structures. Prevention of muscle atrophy and an increase of lean body mass, together with a decrease of body fat and improvements in bone mineral density, as well as increased insulin sensitivity, have all been demonstrated as positive side effects [1]. To ensure an efficient and targeted adaptation with low injury risk, as well as a safe training design, knowledge of the participant specific loading conditions, but also a clear understanding of the external and internal kinematics and kinetics of the resistance exercises themselves, are essential.

Studies have investigated external (e.g. joint moments) [2–5] as well as internal loading conditions (e.g. patello-femoral joint contact forces or muscle activities based on EMG measurements) [6–10] during training exercises such as deadlifts, goodmornings or split squats, resulting in a variety of training recommendations. However, precise and objective comparisons of the actual muscle forces that act throughout these exercises and provide a more complete understanding of the loading patterns for laying the foundations for evidence-based training recommendations, are clearly missing in the literature. Unfortunately, it is not currently possible to measure muscle forces experimentally in a non-invasive manner during exercise performance in vivo. Moreover, data from alternative measurement techniques are not yet sufficient for deducing internal forces and moments for complex dynamic systems such as the lower limbs, in a straight forward manner [11]. Computational models that are able to provide an insight into the internal loading conditions in the human musculoskeletal system [12] have become available using different software packages (e.g. OpenSim SimTK, LifeModeler™, Anybody Modelling System™, Biomechanics of Bodies). Such models are now widely used in clinical and biomechanical gait analysis for studying lower limb dynamics as well as for investigating loading conditions in strength exercises [13–16]. An improved understanding of these muscle and joint contact forces, including not only the magnitude but also the direction of the forces, is essential for appropriate prescription and modification of training exercises, as well as for improving rehabilitation outcomes [13, 17].

Deadlifts, goodmornings and Split squats are all multi-joint resistance exercises used to enhance athletic performance or for reducing the risk of musculoskeletal injury, but also for specific rehabilitation programmes during recovery from injury through targeted improvement of the dynamic stability of lower limb joints [18–20]. To optimize the training results with a reduced risk of injury, desired trained muscle part should be loaded while all the other parts of the body should be unloaded as much as possible. Deadlifts begin with the lifter in a squat position, with arms straight and directed downwards, with an alternating handgrip on the bar [21]. The movement consists of an extension of the knees and hips until the body reaches an upright standing position. Goodmornings start in an upright standing position and, with the barbell on the shoulders, the hips are progressively flexed until maximum hip flexion is reached where the curvature of the lower spine remains in the lordosis, but the knees remain straight throughout. Similarly, split squats are performed with the barbell on the shoulders, but one foot is placed in an anterior position while the participant flexes the knees as far as possible [22]. Deadlifts, goodmornings and split squats are thought to be comparable in their ability to train strength, speed and power in all sport types [23], as well as for improving joint stability in anterior cruciate ligament (ACL) rehabilitation [24, 25], but also with respect to their potential for injury of the lower limbs and the back while performing the exercises [26]. However, the individual internal loading conditions, and specifically the muscle forces that occur during these different exercises remain unknown.

Therefore, with the goal to improve training and rehabilitation designs with respect to specific joint motion, the aim of this study was to examine and compare the specific muscle forces that occur in the lower limbs’ large muscle groups (Hamstrings, Quadriceps and *m gluteus maximus*) as well as total knee joint contact forces, during deadlifts, goodmornings and split squats.

## Methods

The input data used to feed the computational models in this study were published in previous studies examining external loading conditions and joint angles [2, 3]. Two different groups were measured, either performing deadlifts and goodmornings, or split squats. All participants of both groups were sports students and experienced in resistance training (performing strength training two or more times a week). For the deadlifts and goodmornings, 13 participants (4 female, 9 male; 25 ± 4 years, 74 ± 11 kg, 1.80 ± 0.07 m) were observed [2], while five female and six male participants (25 ± 3 years, 68 ± 9 kg, 1.76 ± 0.07 m) were analysed while undertaking split squats [3]. The local ethics committee (Ethics Committee, ETH Zurich, Switzerland) approved both studies (EK2012-N-57 and EK2010-N-24), and participants each provided written informed consent before commencing the testing. To analyse the motion of the body, an opto-electronic system (Vicon, Oxford Metrics Group, UK), recording at 100 Hz with twelve cameras (MX40) was used. Ground reaction forces under each foot were measured using two 400 × 600 mm force plates (type 9281B Kistler, Winterthur, Switzerland) at a frequency of 2 kHz and time synchronised. Additionally, the force plates were calibrated to accurately determine the centre of pressure [27]. The IfB Marker Set consisting of 55 skin markers [28], applied mainly on the lower extremities of the participants was attached by trained personnel. Participants wore their normal training shoes throughout the exercise testing.

After an appropriate warm up of at least 5 Minutes on a stationary bike or a stepper, all participants performed standardised basic motion tasks to functionally determine the centres of rotation (fCoR) and axes in the hips, knees and ankle joints according to List and co-workers [28]. To normalize external and internal loading conditions and be able to compare values across subjects, standardized loads based on subject’s bodyweight (BW) were attached to the barbell rather than loads based on each subject’s repetition maximum one (RM1). An additional load of 25% of each participant’s BW was added to the barbell (deadlifts, goodmornings and split squats) as well as 50% of the participant’s BW for deadlifts only. For the split squats, the step length as well as the frontal tibial angle of the split squats were varied. These variations led to 10 different variations of split squats performed by each subject [3]. For each exercise and exercise variation, more than six repetitions were performed by each participant to be averaged for each subject and each exercise later on. In this study, data from two different studies were used [2, 3] which lead to different subjects performing different strength exercises. Beside the limitation of two different subject cohorts, the population measured in both studies is comparable in respect to gender distribution, age, height, weight, and resistance training experience. Furthermore, in both studies the same relative additional load on the barbell as well as the same data acquisition and processing were used. In order to minimize the influence of the two different groups, all the calculations were performed subject-specific including a functional approach to determine the joint centres (and therefore the length of the segments), gender specific anthropometric data, musculo-skeletal modelling and normalized to body weight.

Captured data were further processed in Matlab (R2014a, MathWorks, Natick, MA, United States) to extract skin marker and joint centre locations in space for each time frame, as well as joint angles and ground reaction forces. Velocity of the barbell markers was used to define the start- and end-point of the strength exercise cycle (v_barbell_ > 40 mm/s respectively <40 mm/s), while the total time of each cycle, also known as lifting time, was normalized to 100%. Using OpenSim (SimTK, Stanford, CA, United States [29]), the extracted data were used to calculate muscle forces in the lower extremities [30]. Here, an adapted standard and widely used musculoskeletal model (‘Gait2392_simbody’ [31–34]) including 14 body segments, 29 degrees of freedom (including 3 DoFs in each knee and ankle joint) and 92 muscles [35] was scaled to each participant’s individual segment length using the fCoRs of the hip, knee and ankle joints, as well as all the skin markers and pre-calculated joint angles. Using the participant’s specific models, the kinematics (in OpenSim termed: inverse kinematics) were calculated as recommended by Schellenberg and co-workers [30], where the fCoRs of the hips, knees and ankles were weighted with a factor of 100, 100 and 60 respectively. Furthermore, all attached skin markers were automatically weighted based on soft tissue artefact [30, 36], with a total weighting of 10 for each segment; pre-calculated joint angles were weighted with 0.02 to avoid flipping of the segments. A standardised OpenSim static optimization, using a cost function that minimised the sum of the squared muscle activation at each time frame, was performed using 6 Hz low pass filtered resultant kinematic data and the measured ground reaction force.

**Fig. 1:**
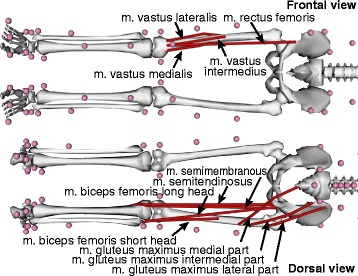
Examined muscles, where the Quadriceps muscles are shown in the frontal plane, while the Hamstrings and *m gluteus maximus* muscles are shown in the dorsal view

Quadriceps, Hamstrings, *m gluteus maximus* muscle groups and absolute total knee joint contact forces were evaluated. Quadriceps consisted of *M. rectus femoris*, *m. vastus lateralis*, *m. vastus medialis*, and *m. vastus intermedius*, while the Hamstrings consisted of *m. biceps femoris* long and short head, *m. semitendinosus* and *m. semimembranosus*. The *m gluteus maximus* muscle was considered to consist of three different parts, the lateral, the intermedial, and the medial part (Fig. 1).

Muscle forces relative to the joint angles and maximal muscle forces during all examined exercises were calculated in each repetition. All muscle forces were then normalised to the participant’s BW and are thus presented in the unit N/kg (i.e. force per kg of bodyweight). Normalized maximal muscle forces are presented for all exercises and all variations. To compare muscle forces and ranges of motion between the different strength exercises, normalized muscle forces were averaged over all participants and plotted as a function of knee and hip flexion angle for deadlifts, goodmornings, and one typical split squats variation. The typical split squat variation was stated using a step length of 70% of the participant’s leg length and a maximal tibial angle of 90°.

For each individual and exercise, maximal muscle forces in all repetitions of an exercise were calculated and averaged over all repetitions. Using this parameter, two statistical tests were performed: First to compare the results between the exercise types and second to compare the results between the different split squat variations. For the first test (comparing between the exercise types), participants’ maximal muscle forces from the deadlifts (25% and 50% additional load), goodmornings (25% additional load), as well as from the typically performed split squat variation (for both the front and rear limbs and using 25% additional load) were used and compared against each other. A linear mixed model with maximal muscle forces and exercise variation as fixed effects and participants as random effects was used to test the influence of the different exercises on each muscle force of the three different muscle groups. For the second test (comparing between the different split squat variations), a linear mixed model with step length and tibial angle as fixed effects and participants as random effects was used to examine maximal muscle forces between the 10 different split squats execution forms. For both investigations, a Bonferroni post-hoc test was conducted and adjusted by means of a Bonferroni correction in all aforementioned cases, if significant differences (*p* < 0.05) were detected. Statistical tests were performed using IBM SPSS (version 22, SPSS AG, Zürich, Switzerland).

## Results

All trials of all participants could be successfully simulated. There was no difference between the two cohorts regarding age, weight and height.

Absolute total knee joint contact forces averaged over all participants ranged from 9 to 111 N/kg for deadlifts with 25%; 12-127 N/kg for deadlifts with 50%; 14-47 N/kg for goodmornings; 14-120 N/kg for all types of split squats in the front limb; 17-65 N/kg for all types of split squats in the rear limb.

### Quadriceps muscles

**Fig. 2:**
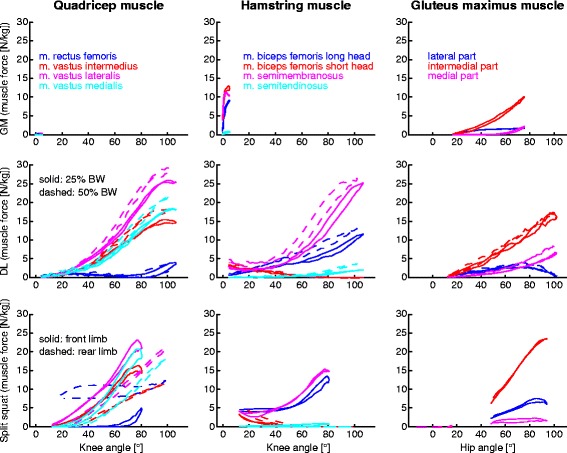
Muscle forces normalised to each participant’s bodyweight as a function of knee and hip flexion angles for the exercises goodmornings (GMs) using 25% of subject’s BW (solid line), deadlifts (DLs) using 25% (solid) and 50% (dashed) of subject’s BW and split squats using a step length of 70% of the participant’s leg length and a maximal tibial angle of 90° for the front (solid) and rear limb (dashed). The four parts of the Quadriceps muscles are shown: *m rectus femoris* in blue; *m. vastus intermedius* in red; *m vastus lateralis* in purple; *m. vastus medialis* in green, together with the four parts of the Hamstring muscles: *m. biceps femoris* long head in blue; *m. biceps femoris* short head in red; *m. semimembranosus* in purple; *m. semitendinosus* in green, as well as the three parts of the *m. gluteus maximus*: lateral part in blue; intermedial part in red; medial part in purple

Different exercises and the different execution form influenced the Quadriceps muscle forces. During goodmornings, almost no forces in the Quadriceps muscles were observed (Table 1, Fig. 2). For deadlifts and the front limb of split squats, forces were similar, and significantly higher (*m. vastus lateralis* and *m. vastus intermedius*) compared to the rear limb of split squats (Table 2). For both exercises, *m. vastus lateralis* showed the highest forces followed by *m. vastus medialis*, *m. vastus intermedius* and *M. rectus femoris* (Table 1). During deadlifts, at knee angles of 40° and above, highly loaded Vasti muscles were observed (Fig. 2). The *M. rectus femoris* force of the rear leg showed a rather constant muscle force (>7 N/kg) over the whole range of motion (RoM, Fig. 1) of the split squats while the vasti forces were significantly lower (Table 1).

**Table 1:** Maximal normalised muscle forces and standard deviation of 11 muscles for 23 different strength exercise variations

				Quadriceps				Hamstrings				*m. gluteus maximus*		
				rf	vi	vl	vm	bl	bs	sm	st	lp	ip	mp
		LL	TA	[N/kg]	[N/kg]	[N/kg]	[N/kg]	[N/kg]	[N/kg]	[N/kg]	[N/kg]	[N/kg]	[N/kg]	[N/kg]
GM 25				1 ± 2	0 ± 1	0 ± 1	0 ± 1	10 ± 3	15 ± 6	14 ± 5	1 ± 1	3 ± 1	12 ± 5	3 ± 2
DL 25				8 ± 5	18 ± 4	30 ± 4	21 ± 5	13 ± 3	6 ± 5	28 ± 5	3 ± 2	6 ± 3	20 ± 4	8 ± 2
DL 50				10 ± 6	22 ± 4	34 ± 6	25 ± 6	15 ± 4	8 ± 5	30 ± 5	5 ± 3	8 ± 3	21 ± 3	11 ± 2
Split Squat 25	front	55%	60°	5 ± 8	22 ± 3	33 ± 3	26 ± 5	12 ± 8	3 ± 2	22 ± 7	2 ± 0	10 ± 3	24 ± 3	4 ± 2
		55%	75°	1 ± 1	21 ± 4	31 ± 4	24 ± 7	12 ± 6	4 ± 2	19 ± 7	2 ± 1	10 ± 4	24 ± 5	3 ± 1
		55%	90°	2 ± 3	19 ± 5	28 ± 7	22 ± 7	12 ± 6	5 ± 3	19 ± 8	1 ± 1	9 ± 5	23 ± 5	3 ± 1
		70%	60°	6 ± 10	22 ± 3	31 ± 5	28 ± 4	17 ± 7	3 ± 2	26 ± 7	2 ± 0	12 ± 3	25 ± 3	4 ± 2
		70%	75°	2 ± 4	21 ± 4	32 ± 4	25 ± 6	14 ± 7	4 ± 2	22 ± 7	2 ± 1	11 ± 3	25 ± 4	3 ± 2
		70%	90°	6 ± 10	20 ± 5	30 ± 6	25 ± 7	16 ± 6	5 ± 2	24 ± 10	2 ± 1	10 ± 4	25 ± 4	4 ± 2
		85%	60°	6 ± 12	22 ± 2	33 ± 4	29 ± 3	21 ± 5	3 ± 2	34 ± 8	3 ± 1	11 ± 4	25 ± 3	7 ± 4
		85%	75°	4 ± 9	21 ± 3	33 ± 5	26 ± 5	16 ± 7	3 ± 2	27 ± 8	2 ± 1	10 ± 3	24 ± 3	4 ± 2
		85%	90°	1 ± 2	20 ± 4	32 ± 6	24 ± 7	16 ± 6	4 ± 3	26 ± 9	2 ± 2	11 ± 3	25 ± 3	4 ± 2
		85%	105°	5 ± 7	17 ± 6	22 ± 8	20 ± 8	16 ± 6	5 ± 3	22 ± 10	1 ± 1	10 ± 4	25 ± 4	4 ± 2
	rear	55%	60°	15 ± 3	11 ± 2	18 ± 3	17 ± 3	0 ± 0	4 ± 2	0 ± 0	0 ± 0	0 ± 0	0 ± 0	1 ± 1
		55%	75°	15 ± 2	12 ± 2	21 ± 5	20 ± 4	0 ± 0	4 ± 2	0 ± 0	0 ± 0	0 ± 0	0 ± 1	1 ± 1
		55%	90°	13 ± 3	14 ± 1	26 ± 6	21 ± 5	0 ± 0	4 ± 2	1 ± 2	1 ± 1	1 ± 0	2 ± 1	2 ± 1
		70%	60°	20 ± 5	8 ± 2	11 ± 4	12 ± 3	0 ± 0	6 ± 3	0 ± 0	0 ± 0	0 ± 0	0 ± 0	0 ± 0
		70%	75°	18 ± 5	10 ± 2	15 ± 5	15 ± 4	0 ± 0	5 ± 3	0 ± 0	0 ± 0	0 ± 0	0 ± 0	0 ± 0
		70%	90°	14 ± 3	12 ± 2	22 ± 5	19 ± 5	0 ± 0	5 ± 2	0 ± 0	0 ± 0	0 ± 0	0 ± 1	1 ± 1
		85%	60°	27 ± 4	5 ± 3	5 ± 5	7 ± 3	0 ± 0	7 ± 5	0 ± 1	0 ± 0	0 ± 0	0 ± 0	0 ± 0
		85%	75°	26 ± 4	7 ± 3	8 ± 6	9 ± 5	0 ± 0	7 ± 4	0 ± 1	0 ± 0	0 ± 0	0 ± 0	0 ± 0
		85%	90°	22 ± 6	11 ± 2	17 ± 5	16 ± 5	0 ± 0	6 ± 3	0 ± 0	0 ± 0	0 ± 0	0 ± 0	0 ± 0
		85%	105°	18 ± 6	12 ± 2	20 ± 6	17 ± 4	0 ± 0	6 ± 3	0 ± 0	0 ± 0	0 ± 0	0 ± 0	0 ± 0

*GM* goodmorning, *DL* deadlifts, *LL* % of participants’ leg length, *TA* tibia angle relative to the ground, *Added weight* additional weight on the barbell as % of participant’s bodyweight. Quadriceps, including *m. vastus lateralis* (vl), *m vastus intermedius* (vi), *m. vastus medialis* (vm) and *M. rectus femoris* (rf); Hamstrings including *m. biceps femoris* short head (bs), *m. biceps femoris* long head (bl), *m. semimembranosus* (sm) and *m. semitendinosus* (st); *m. gluteus maximus* muscles, including three different parts, the lateral part (lp), the intermedial part (ip), and the medial part (mp) were examined

**Table 2:** Significances (*p* < 0.05) between muscle forces of goodmornings with 25% (GM25), deadlifts with 25% (DL25) and 50% (DL50) as well as the front and rear limb of the split squats with a step length equal to 70% of participant’s leg length and a tibia angle of 90° (70% 90° front; 70% 90° rear) with 25% of the participant’s bodyweight as additional load on the barbell

DL25	DL50	70% 90° front	70% 90° rear		
rf, vi, vl, vm	rf, vi, vl, vm	vi, vl, vm	rf, vi, vl, vm	quad	GM25
bs, sm, st	bl, bs, sm, st	bl, bs, sm	bl, bs, sm	ham	
lp, ip, mp	lp, ip, mp	lp, ip	ip	glut	
			vi, vl	quad	DL25
	st		bl, sm, st	ham	
	mp	lp, mp	lp, ip, mp	glut	
			vi, vl	quad	DL50
		st	bl, sm, st	ham	
		mp	lp, ip, mp	glut	
			rf, vi, vl, vm	quad	70% 90° front
			bl, sm	ham	
			lp, ip, mp	glut	

Quadriceps (quad), including *m. vastus lateralis* (vl), *m vastus intermedius* (vi), *m. vastus medialis* (vm) and *M. rectus femoris* (rf); Hamstrings (ham) including *m. biceps femoris* short head (bs), *m. biceps femoris* long head (bl), *m. semimembranosus* (sm) and *m. semitendinosus* (st); *m. gluteus maximus* muscles (glut), including three different parts, the lateral part (lp), the intermedial part (ip), and the medial part (mp) were examined

For the different split squats, vasti muscle forces of the front limb significantly decreased with increasing tibial angle (Tables 1 and 3), while they increased with increasing tibial angle in the rear limb. Additionally, the *m. vastus medialis* force of the front limb increased significantly with step lengths above 55% of leg length. A large step length with a small tibial angle (60°/75°) resulted in a significantly higher *M. rectus femoris* muscle force (Tables 1 and 3). Contrary to the front limb, *m. vastus lateralis* and *m. vastus medialis* of the rear limb increased significantly with an increasing tibial angle and decreased with a larger step.

**Table 3:** significances (*p* < 0.05) between muscle forces of different split squats with different step lengths (55%, 70% and 85% of participant’s leg length) and tibial angles (60°, 75° and 90° tibial angle relative to ground)

Front Limb: Step Length				Rear Limb: Step Length			
70%	85%			70%	85%		
vm	vm	quad	55%	rf, vl, vm	rf, vl, vm	quad	55%
bl, sm	bl, sm, st	ham		bl, bs	bl, bs	ham	
Ip	ip	glut		-	-	glut	
	-	quad	70%		rf, vl, vm	quad	70%
	sm, st	ham			bs	ham	
	-	glut			-	glut	
Front Limb: Tibia Angle				Rear Limb: Tibia Angle			
75°	90°			75°	90°		
vi, vm	vi, vl, vm	quad	60°	vl, vm	rf, vl, vm	quad	60°
bl, sm	bs, sm	ham		-	bl	ham	
-	-	glut		-	-	glut	
	-	quad	75°		rf, vl, vm	quad	75°
	-	ham			-	ham	
	-	glut			-	glut	

Quadriceps (quad), including *m. vastus lateralis* (vl), *m vastus intermedius* (vi), *m. vastus medialis* (vm) and *M. rectus femoris* (rf); Hamstrings (ham) including *m. biceps femoris* short head (bs), *m. biceps femoris* long head (bl), *m. semimembranosus* (sm) and *m. semitendinosus* (st); *m. gluteus maximus* muscles (glut), including three different parts, the lateral part (lp), the intermedial part (ip), and the medial part (mp) were examined. Interactions were observed between mp of the front limb and vi, sm, st, lp, ip and mp of the rear limb

### Hamstrings muscles

During all the examined exercises, the force of *m. semitendinosus* remained low (< 5 N/kg) compared to other muscles, with the highest muscle force observed when performing deadlifts with 50% additional weight (Table 1). The RoMs of the hip and the forces of the other Hamstring muscles were highly influenced by the chosen exercise. During goodmornings, the RoM of the hip remained rather low. Furthermore, *m. biceps femoris* short head, *m. biceps femoris* long head and *m. semimembranosus* muscles were all loaded but only *m. biceps femoris* short head produced a force that was significantly higher than during deadlifts and in the front limb of the split squats. In contrast, *m. biceps femoris* long head and *m. semimembranosus* muscle forces were lower (Tables 1 and 2), but increased with knee flexion angles of >50° during deadlifts and in the front limb of split squats. The muscle forces produced by these two muscles were rather constant at knee angles between 15° and 50° (Fig. 1). Forces in the Hamstring muscle group were almost negligible in the rear limb of split squats, except for *m. biceps femoris* short head at low knee flexion angles of around 20°. Here, the forces were comparable to the front limb and to deadlifts.

A wider stance during split squats increased significantly the force produced by *m. biceps femoris* short head in the rear limb (Table 3). In the front limb, *m. biceps femoris* long head (over all executions: 15.1 ± 6.2 N/kg) and *m. semimembranosus* (average: 23.7 ± 9.5 N/kg) forces were considerable, since these forces were some 3 to 5 times higher than the forces produced by *m. biceps femoris* short head. Both forces increased with an increasing step length and decreasing tibial angle (Table 3).

### Gluteus maximus muscles

Deadlifts and split squats led to significantly more force in the intermedial part compared to goodmornings (Table 2). Furthermore, the intermedial part exhibited higher forces in all exercises (Fig. 2 and Table 1) compared to the medial and lateral parts. Additional weight on the barbell during deadlifts only affected the medial part and therefore led to the highest muscle forces compared to all other exercises.

Similar to the Hamstrings, the *m gluteus maximus* muscles were almost inactive in the rear limb of split squats, not even changing due to different execution variations. Although the value of the intermedial part increased slightly with a wider stance, the *m gluteus maximus* muscle forces were relatively constant over many execution forms of the split squats (Tables 1 and 3). Here, a substantial muscle force increase with increasing hip flexion angle during split squats was observed (Fig. 2).

## Discussion

In this study, three different strength exercises, deadlifts, goodmornings and split squats, with a total of 23 different variations were analysed using musculoskeletal simulation software with the aim to compute internal loading conditions and specifically forces in the muscles. These insights are now able to form the basis for providing evidence-based recommendations for efficient training and rehabilitation.

### Quadriceps muscles

While the largest loading conditions in the Quadriceps were found while performing deadlifts, slightly lower forces were observed while executing split squats. It is therefore reasonable that deadlifts should be favoured if training of the Quadriceps is required, especially if the aim of the training is to strengthen vasti muscles with a desired RoM of approximately 70° to 100° in the knee. Performing split squats should be preferred if activation of vasti muscles or a smaller knee flexion angle (approximately 50° to 80°) is necessary. Since *M. rectus femoris* was only partially recruited in both exercises, but was constantly loaded in the rear leg of the split squats over the whole RoM (20° to 100°), the split squat exercise is recommended to strengthen this muscle, preferably using a large step length and a small tibial angle. Moreover, since loading levels of the different vasti muscles differed in both the front and the rear legs, as well as in different split squat setups (step length and tibial angle relative to the ground), an appropriate split squat setup should be considered for preventing injury and training muscular strength asymmetries within the vasti muscles.

### Hamstrings muscles

Since all exercises examined activated the Hamstrings but during goodmornings the Quadriceps remained inactive, it seems that this exercise has the ability to shift the H:Q ratio towards the Hamstrings, possibly supporting the prevention of, or recovery from, ACL ruptures [2]. However, since low RoMs in the knee were observed during goodmornings compared to split squats and deadlifts, the Hamstrings, especially the one-joint muscles such as *m. biceps femoris* short head, will be trained only at these specific joint angles. The fact that no examined exercise seemed to be able to strengthen *m. semitendinosus* (as indicated by its relatively low levels of force) indicates the requirement of other exercises to train this muscle. However, this phenomenon could result from the low maximal isometric force of the *m. semitendinosus* muscle compared to other Hamstrings muscles.

### Gluteus maximus muscle

Schellenberg and co-workers [2] reported higher external flexion moments in the hip during goodmornings (1.63 ± 0.14 Nm/kg) than during deadlifts (1.4 ± 0.13 Nm/kg) as well as joint moments in the front limb that were comparable to split squats (1.71 ± 0.32 Nm/kg) using the same additional weight on the barbell [3]. Different to the external loading conditions of the hip in the sagittal plane, our data indicate that internal loading of the *m gluteus maximus* forces were significantly lower during goodmornings compared to deadlifts and split squats (Fig. 2). Here, the usage of additional/other muscles seems to be a common recruitment strategy to compensate for the larger external moment [37–39]. This indicates that estimation of the individual muscle loading based on the external moment only, might well lead to result predictions that are incorrect since joint moments alone cannot consider the exercise dependent, individual muscle recruitment. To strengthen the *m gluteus maximus* muscles between a hip flexion angle of approximately 40° and 90° [3], split squats appear to require higher muscular forces and might therefore be a more efficient exercise than deadlifts or goodmornings, while deadlifts should be chosen if larger RoMs in the hip are required.

### Comparison to EMG findings

Training recommendations have recently been provided based on the observed EMG activity [10, 40–45]. For isometric EMG measurements, the quadriceps muscle activation patterns differed across knee flexion angles and between the different muscle parts, especially *m. vastus intermedius*, which was shown to be most sensitive to changes in the muscle length [46]. Regarding the activity of the *m. vastus medialis* and *m. vastus lateralis* in the front limb during split squats, the slightly higher activated medial part is in agreement with previous work [43]. However, through analysing and comparing squats, lunges, step-ups, deadlifts, and leg extensions, Ebben and co-workers [45] reported the highest activity of the biceps femoris during deadlifts. This result is only partially in agreement with our findings. Our data suggest that the short head of the biceps femoris exhibits the highest levels of activity during goodmornings, while the muscle force for the long head of the front limb during split squats was slightly higher than during deadlifts and goodmornings. Since the ability of EMG is known to be limited with respect to assessing the magnitude of muscle forces [46, 47], our findings clearly indicate that rating dynamic strength training exercises based purely on surface EMG measurements may be limited.

### Limitations

This study provides specific loading conditions during different strength exercises and therefore allows the derivation of explicit evidence-based training recommendations. The kinetic and kinematic data reported in our study were gathered during two different measurement sessions with two different groups. However, no differences between the groups were observed, low training loads were used and all models were individualised regarding their anthropometrical data based on the basic motion tasks. Here, especially different segmental lengths between the two groups might influence the external joint moments and therefore also the calculated muscle forces. Please note that comparisons within the different split squat executions and between goodmornings and deadlifts are not affected by this limitation.

The use of musculoskeletal simulation for the determination of internal loading conditions as well as muscle activity and forces suffers from a wide variety of assumptions and simplifications. Here, the participant specific anatomy, physiology, using similar modelling techniques, Schellenberg and co-workers [30] recently showed critical errors in the use of reference musculoskeletal analyses for determining internal loading conditions during squats. An almost linear dependency of the error with knee angle was observed between measured and estimated total joint contact forces, leading to an averaged maximal peak error of approximately 60% at the deepest knee flexion angles. These errors demonstrate the sensitivity of such models for predicting internal loading conditions, suggesting that the results of the current study need to be interpreted with caution. Importantly, however, the authors noted that a comparison of loading conditions across exercises with similar flexion angles should still be possible, but that comparisons across widely ranging joint flexion angles should be interpreted with caution [30]. As a result, the comparative nature of the current study should still allow a reasonable and informative comparison between the different exercises, even though the absolute magnitude of the forces reported is unlikely to be accurate. However, further investigation and validation of the force magnitudes predicted in this study is clearly indicted.

The estimated maximum stress in the muscle tissue of *m. vastus lateralis* was about 61 kPa during the deadlift exercise. This is higher than the commonly acknowledged ultimate stress level of muscle tissue of 50 kPa [48], indicating that the estimated muscle forces are rather higher than those that actually occur physiologically. Here a possible explanation is that the assumed muscle cross section areas of the generic model were smaller compared to our typical participants. Furthermore, it might be that the lever arms of the muscle are underestimated in high flexion positions. Similarly, the reported averaged total joint contact forces ranged between 9 and 127 N/kg, and exceeded the in vivo measured values during deep knee bends (25 N/kg [49]) or squats (26 N/kg [30]). Compared to their cohort, our participants lifted additional weight attached to the barbell, which is also likely to have led to additional synergistic muscle activity (mostly with a shorter lever arm) and therefore to higher total joint contact forces. Please note that during squatting an overestimation of the knee joint contact force occurs only at high knee flexion angles whereas at low knee flexion it is underestimated compared to in-vivo measurements [30]. However, as a comparative study, the differences between the training exercises reported here still contribute new and evidence-based knowledge for informing training and rehabilitation programmes.

### Relevant findings for ACL rupture prevention

One important parameter for the prevention of, or rehabilitation from, ACL rupture seems to be the H:Q ratio [50–53]. Here, amongst others, goodmornings and deadlifts have been used as one of the resistance exercises to shift the H:Q ratio [54]. Under the assumption that the appropriate goal is to shift the ratio towards H, the data presented in this study suggest that goodmornings are a suitable exercise. However, deadlifts and split squats have been shown to shift the ratio rather towards Q and should rather be avoided in an ACL related environment. If the aforementioned flexion dependent error would be taken into account, the H:Q ratio becomes even better during goodmornings, since this particular musculoskeletal model appears to underestimate the internal loading conditions at low knee flexion angles.

## Conclusions

Since specific muscle forces that act during strength exercises were, until now, almost unknown, the results of this study therefore provide coaches and physiotherapists the ability to choose a minimal risk and performance targeted strength-training design for athletes or patients. To reduce the risk of injury of an ACL rupture, performing goodmornings is suggested to shift the H:Q-Ratio towards Hamstrings. Deadlifts produce considerable loading over large ranges of motion in all examined muscles, while only split squats (rear leg) seem to activate the rectus femoris in a substantial manner, but the loading conditions of the exercise itself are highly dependent upon the step lengths and the frontal tibia angle.

### Practical implications

- Muscle loading and ranges of motion are highly dependent on the chosen exercise and execution type.
- Goodmorings should be considered to shift the H:Q-ratio towards H and therefore reduce the risk of injury of ACL-ruptures
- Deadlifts result in large ranges of motion and high loading in the thigh and pelvis muscles.
- By changing step length and angle of the frontal tibia during split squats, specific parts of the thigh and pelvis muscles can be loaded.

## Abbreviations

ACL: Anterior cruciate ligament
BW: Bodyweight
fCoR: Functionally determined centres of rotation
RM1: Repetition maximum one
